# Epigallocatechin Gallate (EGCG): Pharmacological Properties, Biological Activities and Therapeutic Potential

**DOI:** 10.3390/molecules30030654

**Published:** 2025-02-01

**Authors:** Lucia Capasso, Luigi De Masi, Carmina Sirignano, Viviana Maresca, Adriana Basile, Angela Nebbioso, Daniela Rigano, Paola Bontempo

**Affiliations:** 1 Department of Precision Medicine, University of Campania Luigi Vanvitelli, Via L. De Crecchio 7, 80138 Naples, Italy; lucia.capasso2@unicampania.it (L.C.); angela.nebbioso@unicampania.it (A.N.); 2 National Research Council (CNR), Institute of Biosciences and BioResources (IBBR), Via Università 133, 80055 Portici, Italy; luigi.demasi@cnr.it; 3 Department of Pharmacy, School of Medicine and Surgery, University of Naples Federico II, Via Domenico Montesano 49, 80131 Naples, Italy; carmina.sirignano@unina.it; 4 Department of Life Science, Health, and Health Professions, Link Campus University, 00165 Rome, Italy; v.maresca@unilink.it; 5 Department of Biology, University of Naples Federico II, 80126 Naples, Italy; adbasile@unina.it

**Keywords:** epigallocatechin gallate, biological activity, pharmacological activity, preventive activity, human pathologies

## Abstract

Epigallocatechin gallate (EGCG), the predominant catechin in green tea, comprises approximately 50% of its total polyphenol content and has garnered widespread recognition for its significant therapeutic potential. As the principal bioactive component of *Camellia sinensis*, EGCG is celebrated for its potent antioxidant, anti-inflammatory, cardioprotective, and antitumor properties. The bioavailability and metabolism of EGCG within the gut microbiota underscore its systemic effects, as it is absorbed in the intestine, metabolized into bioactive compounds, and transported to target organs. This compound has been shown to influence key physiological pathways, particularly those related to lipid metabolism and inflammation, offering protective effects against a variety of diseases. EGCG’s ability to modulate cell signaling pathways associated with oxidative stress, apoptosis, and immune regulation highlights its multifaceted role in health promotion. Emerging evidence underscores EGCG’s therapeutic potential in preventing and managing a range of chronic conditions, including cancer, cardiovascular diseases, neurodegenerative disorders, and metabolic syndromes. Given the growing prevalence of lifestyle-related diseases and the increasing interest in natural compounds, EGCG presents a promising avenue for novel therapeutic strategies. This review aims to summarize current knowledge on EGCG, emphasizing its critical role as a versatile natural bioactive agent with diverse clinical applications. Further exploration in both experimental and clinical settings is essential to fully unlock its therapeutic potential.

## 1. Introduction

Tea is one of the most popular non-alcoholic beverages consumed worldwide, known not only for its refreshing taste and aromatic qualities, but also for its numerous health benefits. Green tea, particularly favored in Chinese and Japanese cultures, belongs to the Theaceae family and is derived from two main varieties of *Camellia sinensis*: var. *sinensis* and var. *assamica* [1]. It is rich in bioactive compounds, including antioxidants, vitamins, carbohydrates, proteins, minerals, chlorophyll, and polyphenols, which collectively contribute to its well-documented health-promoting effects [2]. The primary bioactive compounds found in green tea leaves are catechins, which make up 25% to 35% of the dry weight of the leaves. These catechins include eight flavonoid-type polyphenolic compounds: (+)-catechin (C), (−)-epicatechin (EC), (+)-gallocatechin (GC), (−)-epigallocatechin (EGC), (+)-catechin gallate (CG), (−)-epicatechin gallate (ECG), (+)-gallocatechin gallate (GCG), and (−)-epigallocatechin gallate (EGCG) [3,4]. Among these, EGCG, a flavonoid-3-ol polyphenol, is the most abundant and is believed to account for approximately 50% of the total polyphenol content in green tea [5].

It is widely regarded as the primary compound responsible for most of green tea’s biological effects [6,7,8,9], owing to its strong antioxidant, anti-inflammatory, and prebiotic properties [10,11]. EGCG is mainly absorbed in the intestine, where the gut microbiota has a fundamental role in its metabolism prior to absorption. The microbiota can break down the large polyphenolic molecules into smaller bioactive metabolites, which in turn enhance EGCG’s systemic effects [12]. Research has indicated that EGCG has favorable bioavailability, as evidenced by its detectable plasma concentrations in fasting individuals after oral consumption [13]. Notably, very little EGCG is excreted in urine, suggesting that it is efficiently transported to the liver or metabolized by the gut microbiota into other bioactive compounds detectable in plasma, bile, and urine [14]. Numerous studies have linked the diverse health benefits of green tea, especially those of EGCG, to its antioxidant and anti-inflammatory abilities [15,16]. EGCG has been shown to influence various physiological pathways, including lipid metabolism and immune function, and may even modulate microbial balance in the gut [17,18,19,20,21]. Given its ability to protect tissues and organs, EGCG has attracted attention for its potential to prevent and treat a wide range of chronic diseases, including cancer, cardiovascular conditions, neurodegenerative disorders, and metabolic diseases [17,22,23,24]. Consequently, the study of EGCG’s therapeutic applications remains of significant scientific interest, with promising implications for improving public health and disease prevention.

This review was systematically prepared by searching for articles and documents from various sources. The research strategy was based on the combination of several keywords, including EGCG, biological activity, pharmacological activity, and preventive activity. By focusing on these terms, we aimed to identify and analyze the multiple roles of EGCG in some of the most common human pathologies. The selected studies highlighted the biological and pharmacological potential of EGCG, particularly its preventive effects through mechanisms such as oxidative stress reduction, lipid metabolism regulation, and epigenetic modulation. This work indicates that EGCG is involved in the regulation of a wide range of cellular signaling pathways through which it can exert its multiple biological activities potentially useful for preventing, mitigating, or slowing the progression of chronic disorders related to the onset and progression of several pathologies, including cardiovascular and neurodegenerative diseases, diabetes, and cancer.

## 2. Chemical–Physical and Pharmacological Properties

**Figure 1:**
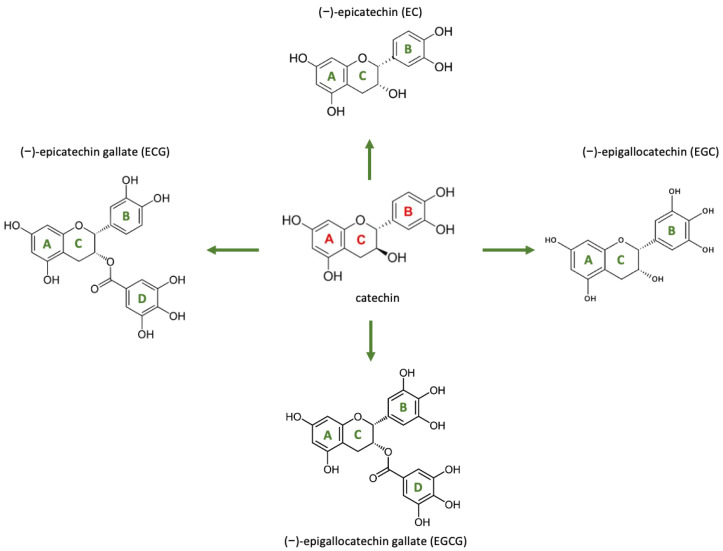
The chemical structures of the four main catechins found in tea and their precursor.

EGCG consists of three aromatic rings (A, B, and D) interconnected by a pyran ring (C), as depicted in Figure 1. In its pure form, it appears as a white or pale pink odorless powder or crystalline substance. EGCG is soluble in solvents such as water, ethanol, methanol, acetone, tetrahydrofuran, and pyridine, with a melting point of 218 °C (Alexis Corporation, Hangzhou, China; Enzo Life Sciences, Farmingdale, NY, USA; Sigma-Aldrich, St. Louis, MO, USA). Its unique, easily modifiable chemical structure is primarily responsible for its biological activities and health benefits.

Catechins, including EGCG, exhibit antiradical properties through the oxidation of phenolic groups in the B and D rings via atomic or single-electron transfer, producing quinones and semiquinones [4,25]. Structural modifications to these rings influence proteasome inhibition, as demonstrated by Landis-Piwowar et al., who reported that dehydroxylation of the B and/or D rings reduces proteasome inhibitory activity in vitro. Interestingly, this activity is retained by peracetate-protected analogs, which also induce apoptosis in tumor cells. These findings highlight the potential of B-ring/D-ring peracetate-protected EGCG analogs as promising candidates for anticancer drug development [26]. Further structure–activity relationship studies have revealed that phenolic groups on ring A enhance the inhibition of heat shock protein 90, whereas those on ring D are less favorable for this activity [27]. Additionally, the presence of a 5′ hydroxyl group on ring B significantly increases urease inhibition (35–104 times higher) compared to catechins lacking this group and has shown efficacy against *Helicobacter pylori* growth in the stomach [28].

### 2.1. Main Pharmacokinetic Properties

The pharmacokinetics of EGCG and green tea polyphenols have been thoroughly investigated in both animal models [29,30,31,32] and human studies [33,34,35]. EGCG is absorbed quickly in the gastrointestinal tract, then distributed throughout the body, metabolized primarily in the liver and colon, and undergoes enterohepatic recirculation, enabling partial reabsorption. Its metabolites are eliminated through bile and urine, with only trace amounts of unchanged EGCG detectable in the urine after oral intake [14]. Despite extensive research, pharmacokinetic parameters show significant variability between experiments and among individuals. Factors such as fasting, storage conditions, albumin levels, vitamin C, fish oil, and piperine have been shown to affect plasma concentrations and the overall bioavailability of EGCG. Conversely, bioavailability is reduced by processes such as air oxidation, sulfation, glucuronidation, gastrointestinal degradation, and interactions with Ca^2+^, Mg^2+^, and trace metals, as well as by genetic variations like COMT polymorphisms [8]. EGCG’s oral bioavailability is generally low, with marked differences observed across species, for example, bioavailability rates of 26.5% in CF-1 mice and just 1.6% in Sprague Dawley rats [36].

Following oral administration, EGCG is rapidly absorbed, reaching peak plasma concentrations approximately one hour post-intake [34]. The limited bioavailability of catechins may be attributed to significant gastrointestinal metabolism and hepatic extraction shortly after absorption [31]. A pharmacokinetic study in healthy individuals receiving single doses of EGCG (ranging from 50 mg to 1600 mg) revealed that plasma concentrations exceeded 1 μM only when doses of 1 g or higher were administered. Specifically, a dose of 1600 mg yielded a Cmax of 3392 ng/mL (range: 130–3392 ng/mL), with peak levels observed between 1.3 and 2.2 h, AUC (0–∞) values ranging from 442 to 10,368 ng·h/mL, and a half-life (t1/2z) of 1.9 to 4.6 h. Further evaluations of EGCG plasma and safety parameters over four weeks using once-daily and twice-daily regimens demonstrated variable serum levels, with an oral bioavailability of 20.3% relative to intravenous administration. Doses between 800 and 1600 mg were deemed safe and well-tolerated [30,34].

Studies on the distribution of EGCG have revealed that, despite its limited absorption, it is rapidly disseminated throughout the body or quickly converted into metabolites. The concentrations of EGCG detected in tissues represent only 0.0003–0.45% of the total ingested amount [37]. EGCG and its metabolites have been identified in biological fluids such as serum, plasma, saliva, feces, and urine, as well as in various organs and tissues, including the liver, kidneys, intestinal mucosa (small intestine and colon), prostate, tumor tissues, fetuses, and placenta. Additionally, EGCG can cross the blood–brain barrier, allowing it to reach the brain [30,31,35].

**Figure 2:**
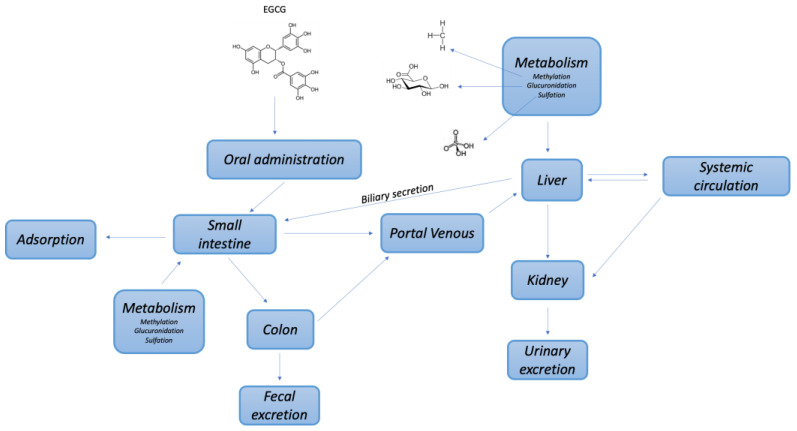
Absorption and metabolism of EGCG.

EGCG and other catechins undergo enzymatic metabolism in the human body, producing various biologically active metabolites (Figure 2).

The primary metabolic pathways for tea catechins include methylation, glucuronidation, sulfation, and ring fission biotransformation. Catechol-*O*-methyltransferase (COMT) is a key enzyme that breaks down catechol-like structures, including dietary phytochemicals, to remove potentially toxic or active compounds [3]. Methylated catechin derivatives have been identified in rat liver homogenates. Glucuronidation, catalyzed by UDP-glucuronosyltransferases (UGT), enhances water solubility and facilitates the excretion of both endogenous and exogenous compounds through urine or feces. The main glucuronidated metabolite of EGCG is EGCG-4″-*O*-glucuronide [38]. Sulfation, mediated by sulfotransferase (SULT) enzymes, involves transferring a sulfate group to alcohol or amine substrates [3]. LC/MS analyses have identified sulfated forms of EC, EGC, and EGCG in human and rodent samples [3,38]. Additionally, glucosidation at the 7-position of ring A and the 4′ position of ring B results in the formation of a novel metabolite, 7-*O*-beta-d-glucopyranosyl-EGCG-4″-*O*-beta-d-glucopyranoside, detected using the LC/ESI-MS2 method. Thiol conjugation processes lead to mono-, bi-, and triglutathione conjugates of catechin dimers, formed via reactions with glutathione and quinone derivatives. Furthermore, microbial catabolism by gut microbiota plays a crucial role in transforming polyphenols into bioactive metabolites, contributing to the health benefits associated with tea consumption [3]. EGCG is excreted mainly through bile, while EGC and EC are excreted through both bile and urine [29,35].

### 2.2. Toxicity

Toxicity studies in rats have shown no significant changes in body weight, hematological, or biochemical parameters following oral administration of 15 or 75 mg/kg of green tea extract (GTE) for 28 days. Similarly, when EGCG was administered via gastric tube at daily doses of 0, 45, 150, or 500 mg/kg body weight over consecutive days, no adverse effects were observed [39]. Other studies have reported no toxicity following the dietary administration of EGCG up to 500 mg/kg/day, establishing a no-observed-adverse-effect level (NOAEL) for EGCG at 500 mg/kg/day [40].

No teratogenic or reproductive toxic effects were observed in rats after administering EGCG during organogenesis at doses of 1000 mg EGCG/kg/day or when plasma concentrations reached 191 μg/mL [41].

Isbrucher et al. found no evidence of genotoxicity in rats following oral administration of EGCG at doses of 500, 1000, or 2000 mg/kg, or intravenous injections of 10, 25, or 50 mg/kg/day. These doses, resulting in significantly higher plasma concentrations, did not induce genotoxic effects [42].

There are no epidemiological studies or case reports linking EGCG exposure to an increased cancer risk in humans [43]. EGCG is not classified as a carcinogen. On the contrary, due to its widespread presence in nature and its low toxicity, EGCG is considered a promising candidate for anticancer therapy [43]. It has been shown to exert antitumor effects against several human carcinomas and tumor cell lines, with its antiproliferative, pro-differentiating, and apoptosis-inducing properties suggesting it could be a valuable therapeutic agent in cancer treatment [44,45,46,47].

There is evidence in the literature describing specific target organ toxicity, particularly liver toxicity, which is associated with altered liver enzyme function. Several studies have documented liver damage linked to green tea consumption [48,49,50,51,52,53]. Mazzanti et al. examined the causal relationship between green tea and liver injury. Laboratory tests revealed elevated levels of transaminases, gamma-glutamyl transpeptidase, alkaline phosphatase, and bilirubin. Liver damage was classified as predominantly hepatocellular (62.50%), cholestatic (18.75%), or mixed (18.75%) [54]. High doses of EGCG (500 and 1000 mg/kg/day) have also been shown to induce cardiac fibrosis in mice, an effect likely related to the inhibition of AMPK-dependent signaling pathways. It is important to note that these toxic effects occur at doses much higher than those typically achieved through regular tea consumption, though they may be easily reached when consuming concentrated tea-based food supplements [55].

Numerous studies highlight the potential benefits of EGCG in combating various human diseases, including cancer, diabetes, obesity, cardiovascular conditions, and neurodegenerative disorders [22,56].

Despite its promising therapeutic and preventive properties, EGCG’s clinical application is limited by its poor intestinal absorption and inherent instability. To address these limitations, the encapsulation of EGCG in nanocarriers has significantly improved its stability and therapeutic efficacy [57]. Importantly, EGCG’s non-toxic nature and lack of reported side effects make it an appealing candidate for disease prevention and treatment research. Research has demonstrated that nanocarriers, particularly lipid-based and polymeric systems, enhance the bioavailability and stability of EGCG due to their biocompatibility [58]. Other nanocarrier systems, including liposomes, gold nanoparticles, inorganic materials, proteins, and peptides, have also been explored [59,60]. However, concerns remain about the safety of gold and inorganic nanocarriers. Promising results have been observed when nanocarriers were modified with mucoadhesive ligands, such as chitosan, which facilitate intestinal absorption by opening tight junctions in the intestinal barrier [61,62]. Additionally, functionalizing the surface of nanocarriers with targeting ligands can enhance the selective delivery of EGCG to abnormal or tumor cells, improving therapeutic outcomes. Overall, the literature suggests that nanoformulations hold great potential for enhancing EGCG’s therapeutic activity and overcoming its current limitations [61].

### 2.3. Interactions, and Synergistic Adverse Effects

The bioactive compounds in tea can interfere with the absorption of various molecules. For instance, an interaction with the folate transporter has been reported, leading to reduced bioavailability of folic acid [63]. Therefore, it is advised that pregnant women, individuals with megaloblastic anemia, or those for whom folic acid reduction may have clinical consequences avoid the concurrent consumption of green tea and folic acid. EGCG has also been shown to inhibit the intestinal absorption of non-heme iron in a dose-dependent manner in a controlled clinical trial. Several epidemiological studies have suggested that tea consumption may adversely affect iron status, though the results are inconsistent. Published studies indicate that black tea reduces iron absorption more significantly than green tea [39].

The use of EGCG has the potential to enhance the effects of conventional cancer therapies through additive or synergistic actions, as well as by ameliorating the harmful side effects of these treatments [64,65].

After treatment with green tea, no significant changes in hematological or biochemical parameters have been observed. The most commonly reported side effects are mild, including nausea, stomach discomfort, abdominal pain, and headaches [34].

## 3. Biological and Therapeutic Potential of EGCG

**Figure 3:**
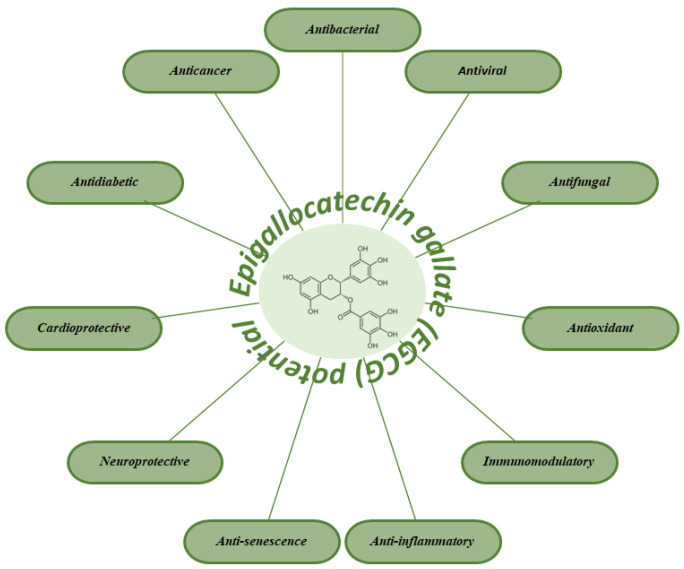
Biological and therapeutic potential of EGCG.

Numerous preclinical studies, including both in vitro and in vivo experiments, as well as clinical trials, have highlighted the broad spectrum of biological and pharmacological properties exhibited by polyphenolic compounds, particularly EGCG. These properties include antimicrobial activity [66], anti-carcinogenic (anti-tumorigenic) effects [67], antioxidant activity, anti-allergic effects [68], anti-inflammatory properties [69], anti-diabetic effects [70], anti-hypercholesterolemic (lipid clearance) effects [71], anti-atherosclerosis, anti-hypertensive properties [71], anti-aging effects [72], a reduced risk of osteoporotic fractures [73], neuroprotective effects [74], cardioprotective benefit [68], and immunomodulatory activity (Figure 3) [75]. The biological activity of green tea polyphenols, and EGCG in particular, is closely linked to their pharmacokinetic properties and bioavailability [76]. The effects of EGCG result from a variety of mechanisms. EGCG can interact directly with proteins and phospholipids of membrane, stimulating intracellular signaling pathways [77]. Additionally, once transported to intracellular compartments such as the cytosol, mitochondria, lysosomes, and nuclei, EGCG can mediate further biological actions. The specific biological effects of EGCG can vary based on several factors, including cell type, stress conditions, and the concentration of EGCG [78].

### 3.1. Antibacterial Activity of EGCG

The antimicrobial properties of EGCG have been well documented in numerous studies [66]. EGCG demonstrates significant antibacterial activity against various bacterial strains, including methicillin-resistant *Staphylococcus aureus* (MRSA), *Stenotrophomonas maltophilia*, *Mycobacterium tuberculosis*, *Helicobacter pylori*, and *Streptococci* spp. Additionally, EGCG enhances the effectiveness of beta-lactam antibiotics, such as methicillin. An in vitro study evaluating the antibacterial potential of EGCG against clinical isolates of *S. aureus* showed that EGCG, due to its negative charge, binds to positively charged lipids in the bacterial cell membrane. This binding causes structural damage to the membrane, leading to the intramembranous leakage and fragmentation of the lipid bilayer. Furthermore, EGCG has been shown to inhibit the activity of bacterial-cell-membrane peptidoglycan penicillinase, either directly or indirectly, resulting in the reduction or inhibition of biofilm production by *S. aureus* [9]. Catechins, including EGCG, inhibit bacterial DNA gyrase, a crucial enzyme for bacterial survival, by binding to the ATP-binding site of the gyrase B subunit [79]. In addition, EGCG has anti-folate activity, inhibiting dihydrofolate reductase, an enzyme that converts 7,8-dihydrofolate to 5,6,7,8-tetrahydrofolate, leading to the termination of DNA synthesis [9]. Steinmann et al. demonstrated that EGCG induces cell death in *S. pyogenes* and inhibits bacterial attachment to human cells [66]. Moreover, EGCG has shown cytoprotective effects against *H. pylori*-induced gastric cytotoxicity by interfering with Toll-like receptor 4 (TLR-4) signaling, which is triggered by *H. pylori* infection [80].

### 3.2. Antiviral Activity of EGCG

EGCG has demonstrated inhibitory activity against a wide range of viral families, including Retroviridae, Orthomyxoviridae, Flaviviridae, and Retroviridae, which encompass several important human pathogens, such as influenza viruses, enteroviruses, adenoviruses, human immunodeficiency virus (HIV), Epstein–Barr virus (EBV), herpes simplex virus (HSV), hepatitis B virus (HBV), and hepatitis C virus (HCV) [66,81]. Unlike its antibacterial effects, which require high concentrations, EGCG has shown significant antiviral activity at physiological concentrations in many of these studies [66]. GTE inhibits the infectivity of influenza viruses, and EGCG specifically inhibits Influenza A (IAV) and B (IBV) viruses by preventing their adsorption to host cells [81,82]. EGCG and epicatechin gallate (ECG) have been identified as potent inhibitors of influenza virus replication in MDCK cell cultures, with effects observed across all tested subtypes of influenza, including A/H1N1, A/H3N2, and B. EGCG, in particular, demonstrated superior hemagglutination inhibition activity compared to other catechins [83]. EGCG also exerts antiviral effects against adenoviruses of the Adenoviridae family by directly inactivating viral particles, inhibiting viral growth, and preventing the activity of viral protease [84]. Furthermore, a single-dose administration of EGCG (ranging from 50 to 1600 mg) has been shown to inhibit HCV replication, with no reported adverse effects in human volunteers [85]. EGCG effectively inhibited the secretion of HBV-specific antigens (HBsAg, HBeAg) and demonstrated stronger antiviral activity than lamivudine in a dose- and time-dependent manner [86]. At concentrations above 50 μM, EGCG also suppressed the expression of EBV lytic proteins, thereby blocking the virus’s lytic cycle [87]. Furthermore, EGCG interferes with the HIV life cycle by preventing the attachment of HIV-1 virions to host cells [88], inhibiting an early stage of viral entry by preventing the virus from anchoring to the cell surface [89], and blocking reverse transcriptase, the enzyme responsible for converting RNA into DNA, thereby inhibiting viral replication [90]. For HBV, a hepatotropic DNA virus that contributes to cirrhosis and hepatocellular carcinoma (HCC) [91], EGCG inhibits HBV-DNA replication and reduces the levels of hepatitis B surface antigen (HBsAg) mRNA in HBV-infected hepatocytes [92]. This effect is mediated through the downregulation of hepatocyte nuclear factor 4α (HNF4α) via extracellular signal-regulated kinase (ERK) signaling [93,94]. Additionally, EGCG prevents HBV entry into hepatocytes by modulating the endocytosis and degradation of the sodium taurocholate co-transporter polypeptide (NTCP), a key receptor for HBV infection. By regulating NTCP, EGCG effectively blocks a significant portion of HBV infection [95]. Previous studies have also demonstrated that EGCG can mediate lysosome acidification in hepatocytes, disrupting the formation of incomplete autophagosomes required for HBV replication [96]. Beyond HBV, EGCG exhibits notable activity against HCV, another major pathogen responsible for HCC [97]. In particular, EGCG boosts antiviral innate immunity in hepatocytes by promoting the expression of toll-like receptor 3 (TLR3), retinoic acid-inducible gene I (RIG-I), and interferon lambda 1 (IFN-λ1) [98,99]. EGCG has been shown to modify the structure of HCV viral particles, decreasing their ability to adhere to hepatocytes [100,101], and may involve HCV envelope or capsid proteins in this process [102,103]. Moreover, EGCG inhibits CD81 receptors, which are critical for HCV entry into hepatocytes, by upregulating specific miRNAs such as miR-548m and miR-194, which reduce HCV infectivity in Huh7 cells [104,105]. EGCG is also more effective than embelin, a known inhibitor of HCV NS3/4A protease, an enzyme essential for viral replication and maturation [106]. In summary, EGCG shows great potential as a natural antiviral agent, particularly against liver-associated viruses, and its combination with existing antiviral therapies could offer a novel therapeutic approach for treating viral hepatitis and liver cancer, as well as other viral infections.

### 3.3. Antifungal Activity of EGCG

The antifungal potential of EGCG has been primarily investigated against yeast strains such as *Candida* spp. (*C. albicans*, *C. glabrata*) and dermatophytes (*Trichophyton mentagrophytes*, *T. rubrum*). Studies have revealed several key mechanisms underlying its antifungal activity. These include the disruption of osmotic integrity [107] and anti-folate activity through the inhibition of dihydrofolate reductase (DHFR), an enzyme critical for the synthesis of purines, pyrimidines, and amino acids, which consequently impairs fungal cell growth [108]. EGCG has also been shown to hinder biofilm formation, both structurally and metabolically. Further supporting these findings, in vitro studies have demonstrated morphological changes in *T. mentagrophytes* isolates upon treatment with EGCG, including deformation, swelling, granular accumulation, and inhibition of hyphal growth [109,110]. Additionally, EGCG has shown synergistic antifungal effects when combined with traditional antifungal drugs, particularly against *C. albicans* [111]. These results highlight EGCG’s potential as an effective antifungal agent, especially when used in combination with other therapeutic agents.

### 3.4. Antioxidant Effects

Antioxidation plays a crucial role in maintaining human health by protecting cells from oxidative stress. The antioxidant properties of tea catechins, particularly EGCG, have been widely recognized in both in vitro and in vivo studies. Due to its chemical structure, EGCG is classified as an effective antioxidant compound. The phenolic rings in EGCG act as electron traps and free radical scavengers [112,113], inhibiting the formation of reactive oxygen species (ROS) and mitigating oxidative damage. EGCG has been shown to scavenge a wide range of radicals, including superoxide, hydroxyl [78,114,115,116,117], and peroxyl radicals [118], as well as other reactive species such as nitrogen oxide (NO) [119], lipid free radicals, carbon-centered radicals, and singlet oxygen [116,120]. In addition, EGCG is capable of scavenging peroxynitrite, a highly reactive molecule that can cause tyrosine nitration in blood platelets, thus reducing oxidative stress-related damage [121,122]. Its antioxidant properties also extend to mitochondrial protection, where it helps improve mitochondrial function [123]. Beyond radical scavenging, EGCG also exhibits metal-chelating properties. These are attributed to specific structural elements in EGCG, such as the ortho-3′,4′-dihydroxy moiety and the 4-keto, 3-hydroxyl or 4-keto, 5-hydroxyl moieties, which prevent the generation of free radicals through metal ion catalysis [124,125]. Like other flavonoids, EGCG can bind to and inactivate transition metal ions, thereby inhibiting the Fenton reaction, which is a major pathway for ROS formation [126]. Other mechanisms by which EGCG exerts its antioxidant effects include electron transfer to ROS-induced radical sites on DNA and the formation of stable semiquinone free radicals. Studies have shown that the antioxidant effects of catechins, including EGCG, are more pronounced than those of vitamins C and E [120,127,128,129,130]. Moreover, EGCG has been shown to protect cells from ROS-induced damage caused by various agents, including hydrogen peroxide [114,125], primaquine [125], and iron [125,126]. However, at high concentrations, EGCG can exhibit pro-oxidant activity by inducing autoxidation, which generates hydroxyl radicals, hydrogen peroxide, and quinone intermediates, leading to cytotoxicity [131,132,133,134]. For instance, the autoxidation of EGCG can produce catecholquinone, which binds to erythrocyte membrane proteins, leading to protein aggregation and membrane damage [135]. This instability is particularly pronounced at alkaline pH and can be influenced by factors such as pH, temperature, metal ions, and EGCG concentration [136]. At physiological concentrations (from 1–2 μM to 10 μM), EGCG generates low levels of ROS, which can activate cellular protective pathways, thereby promoting its antioxidant effects while maintaining cellular homeostasis [134,137].

### 3.5. Anti-Inflammatory, Immunomodulatory, and Antifibrotic Effects

The inflammatory response is a complex process involving the recruitment and activation of immune and inflammatory cells, the release of pro-inflammatory cytokines, and the generation of reactive oxygen and nitrogen species (ROS/RNS). These events are largely driven by the activation of transcription factors like NF-κB and AP-1, which translocate to the nucleus and upregulate genes involved in inflammation and tissue damage. NF-κB, in particular, plays a pivotal role in regulating cellular processes, including inflammation, immune responses, cell growth, and oxidative stress responses. EGCG has been widely recognized for its anti-inflammatory effects, primarily through its regulation of key signaling pathways. In multiple in vitro and in vivo studies, EGCG has been shown to suppress NF-κB activation, inhibit its nuclear translocation, and block AP-1 activity [138,139]. This leads to the downregulation of pro-inflammatory enzymes like iNOS and COX-2 and scavenging of ROS/RNS, including nitric oxide and peroxynitrite [140]. Notably, EGCG also reduces the release of inflammatory mediators, demonstrating its potential to mitigate inflammatory responses [141]. For instance, studies on normal human epidermal keratinocytes revealed that EGCG modulates NF-κB activity and protects cells from UV-induced damage, suggesting its role as a photoprotective agent [142]. Furthermore, in animal models of intestinal inflammation, EGCG alleviated mucosal injury by modulating NF-κB and other related genes. In a rat model of ulcerative colitis, EGCG administration reduced disease severity by targeting the TLR4/MyD88/NF-κB pathway [141,143]. Similarly, EGCG has been shown to suppress airway inflammation by reducing IL-8 release, a cytokine involved in neutrophil aggregation and ROS production. Additionally, EGCG blocks the JAK1/2 signaling pathway, thereby reducing pro-inflammatory gene expression mediated by P2X4 receptors in endothelial cells [144]. Interestingly, while EGCG is primarily known for its anti-inflammatory effects, conflicting evidence exists regarding its immunomodulatory properties. Some studies indicate EGCG reduces the release of tumor necrosis factor (TNF), decreases leukocyte infiltration, and alleviates UV-induced inflammation, while others suggest pro-inflammatory effects, such as enhanced interleukin-1 production and B-cell mitogenic activity. This duality may be context-dependent and warrants further investigation [145]. Beyond its anti-inflammatory properties, EGCG also exhibits antifibrotic effects, particularly through its regulation of NF-κB signaling. Fibrosis, a pathological outcome of chronic inflammation, involves excessive extracellular matrix deposition that can lead to organ dysfunction, such as liver cirrhosis, myocardial fibrosis, or peritoneal fibrosis [146,147,148]. EGCG has demonstrated the ability to inhibit hepatic stellate cell activation and proliferation, reduce collagen synthesis, and downregulate PDGFR and IGF-1R gene expression—both critical factors in liver fibrosis progression [146,149,150]. It has also been shown to reduce MMP-2 mRNA expression, thereby limiting matrix remodeling and further fibrosis development [146]. In cardiac fibroblasts stimulated with AngII, EGCG suppressed collagen synthesis and fibronectin expression by inhibiting NF-κB translocation and downregulating CTGF gene expression [151]. Similar antifibrotic effects have been reported in peritoneal fibrosis, where EGCG reversed fibrotic changes by suppressing NF-κB activity [152]. In addition to preventing fibrosis, EGCG exhibits anti-collagenase properties. Collagenase, an enzyme that hydrolyzes collagen’s helical structure, compromises collagen integrity and function. EGCG has been shown to stabilize collagen and protect it from enzymatic degradation [153,154]. Madhan et al. hypothesized that hydrogen bonding and hydrophobic interactions between EGCG and collagenase inhibit its activity, making collagen resistant to breakdown [155]. Overall, EGCG exerts multifaceted biological effects through its ability to regulate signaling pathways, suppress inflammatory mediators, and protect extracellular matrix components. Its anti-inflammatory, antifibrotic, and collagen-stabilizing properties make it a promising therapeutic agent for various chronic inflammatory and fibrotic diseases, further underscoring its importance in human health.

### 3.6. Anti-Senescence Activity

Senescence is a critical biological process involved in embryogenesis, tissue regeneration, aging, and the progression of various diseases, including cardiovascular, renal, and hepatic conditions, as well as cancer [156,157]. Recent research has highlighted the potential of EGCG to modulate cellular senescence, demonstrating its protective effects against oxidative stress and age-associated cellular changes [158,159]. For instance, Shin et al. reported that EGCG can mitigate oxidative stress-induced senescence in human mesenchymal stem cells (hMSCs) exposed to H_2_O_2_. This effect was attributed to the downregulation of the p53-p21 signaling pathway and the enhanced expression of Nrf2, a key regulator of antioxidant defense mechanisms [159]. Comparable results were observed in primary cells such as rat vascular smooth muscle cells (RVSMCs), human dermal fibroblasts (HDFs), and human articular chondrocytes (HACs). EGCG, administered at concentrations of 50 and 100 μM, effectively delayed cellular senescence and restored cell-cycle progression, protecting cells from oxidative damage [160]. In addition, studies conducted on 3T3-L1 preadipocytes revealed that EGCG prevented senescence induced by oxidative stress through the regulation of PI3K/Akt/mTOR and AMPK pathways. This resulted in reduced ROS production, decreased activity of iNOS and COX-2, inhibition of NF-κB and p53 signaling, and the suppression of cell-cycle arrest. EGCG also promoted apoptosis by downregulating the anti-apoptotic protein Bcl-2 [161]. Similarly, in WI-38 fibroblasts, EGCG concentrations ranging from 25 to 100 μM reduced the levels of TNF-α, IL-6, and ROS while enhancing the expression of E2F2 and superoxide dismutases (SOD1 and SOD2), enzymes vital for cellular antioxidant defense. EGCG’s ability to modulate the retinoblastoma protein pathway further highlights its role in delaying senescence and improving cellular resilience against oxidative damage [162]. Collectively, these studies underscore EGCG’s potential to counteract cellular senescence through various mechanisms, including oxidative stress reduction, DNA damage repair, and cell-cycle regulation. Despite the promising preclinical data, further investigation is essential to fully elucidate its therapeutic potential in age-associated disorders. In particular, the senolytic properties of EGCG, which may alleviate aging-related conditions, warrant additional clinical studies to confirm its efficacy and long-term safety.

### 3.7. Anticancer Activity

**Figure 4:**
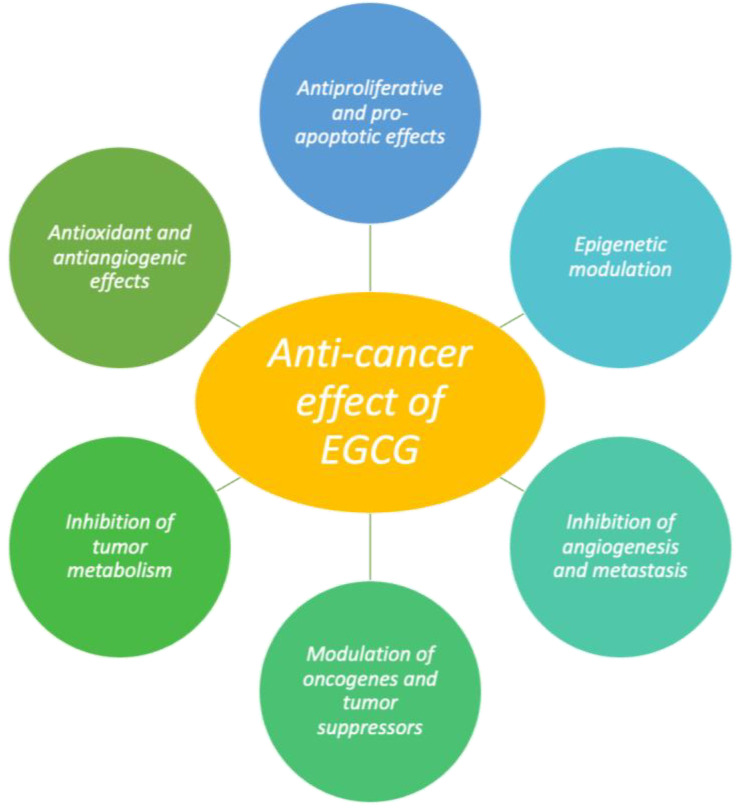
EGCG in cancer: potential for chemoprevention and therapy.

EGCG has garnered significant attention for its potential anticancer properties, with extensive research demonstrating its ability to prevent and treat various forms of cancer. Its effects are multifaceted, involving complex interactions with multiple signaling pathways and biological mechanisms critical to cancer development and progression [163,164]. Studies have shown that EGCG exhibits antitumor effects across a range of cancers, including breast, prostate, lung, colorectal, melanoma, as well as hematological cancers like acute myeloid leukemia, chronic myeloid leukemia, and multiple myeloma [67,164,165,166,167,168,169,170,171]. In prostate cancer cells, EGCG induced dose-dependent apoptosis, and in breast cancer cells, it inhibited cyclin-dependent kinases, blocking cell-cycle progression; in human breast cancer cells, the inhibition of cyclin-dependent kinases by EGCG blocked cell-cycle progression [67,172,173,174]. More recent findings have supported these results, showing EGCG’s ability to induce apoptosis in breast cancer cells in a time- and dose-dependent manner without significant toxicity to normal cells [171]. Furthermore, EGCG has been shown to activate key apoptotic pathways, such as caspase-3 activation, cytochrome c release, and PARP cleavage, in various cell models, including PC12 cells exposed to oxidative stress [175]. Additionally, EGCG influences several molecular pathways involved in cancer progression and prevention. Granja et al. identified key mechanisms through which EGCG exerts its anticancer effects: (1) the inhibition of DNA hypermethylation by blocking DNA methyltransferase (DNMT); (2) the repression of telomerase activity; (3) the suppression of angiogenesis via the inhibition of HIF-1α and NF-κB; (4) the prevention of cellular metastasis by inhibiting matrix metalloproteinases (MMPs); (5) the promotion of apoptosis through the activation of pro-apoptotic proteins like BAX and BAK while downregulating anti-apoptotic proteins like BCL-2 and BCL-XL; (6) the upregulation of tumor suppressor genes such as p53 and PTEN; (7) the inhibition of inflammation and proliferation via NF-κB suppression; and (8) anti-proliferative activity through the modulation of MAPK and IGF1R pathways (Figure 4) [163].

**Figure 5:**
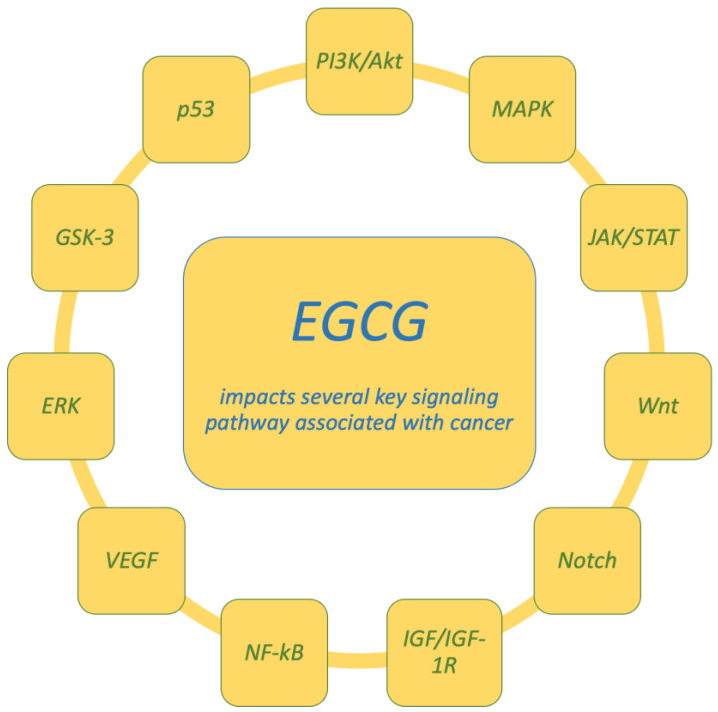
Role of EGCG in signaling pathways involved in cancer.

EGCG has also been demonstrated to prevent cancer-cell invasion and migration via a number of routes, including autophagy, lysosomal membrane permeabilization-mediated cell death, and caspase-dependent and caspase-independent apoptosis. JAK/STAT, MAPK, PI3K/AKT, Wnt, and Notch are among the signaling pathways that predominantly mediate the actions of EGCG (Figure 5) [146]. For example, EGCG has been shown to prevent liver tumorigenesis induced by diethylnitrosamine by inhibiting the IGF/IGF-1R axis, reducing chronic inflammation, and improving hyperinsulinemia [176]. Moreover, EGCG inhibits hepatocyte growth factor (HGF), which is involved in tumor migration and invasion [177,178]. EGCG’s antitumor effects are further facilitated by its interaction with the 67 kDa laminin receptor (67LR), a receptor overexpressed in various cancers, including breast, pancreatic, and gastric cancers, as well as melanoma and multiple myeloma. EGCG’s ability to modulate 67LR activity correlates with its ability to inhibit tumor progression and enhance the prognosis in these cancers [179]. Additionally, EGCG has shown potential in modulating epigenetic changes, particularly through DNA methylation and histone modifications. It inhibits DNMT activity, leading to the reactivation of tumor suppressor genes such as p16 (INK4a), RAR, MGMT, and hMLH1 [67,180,181]. EGCG’s role in DNA hypomethylation has been observed in both in vitro and in vivo models, where it enhances the expression of genes like PTEN and CDKN1A, thereby promoting apoptosis and inhibiting cell proliferation. EGCG has also been shown to influence the expression of tissue inhibitors of metalloproteinases (TIMPs) and MMPs, which are involved in tumorigenesis [182]. EGCG has demonstrated potential in clinical applications as well, with studies showing its ability to modulate TIMP-3 levels in prostate cancer patients, suggesting a role in therapeutic cancer treatment [182]. Furthermore, EGCG’s topical application in a mouse model of photocarcinogenesis reduced tumor incidence and size, likely through its inhibition of UVB-induced DNA hypomethylation and modulation of DNMT and histone deacetylase (HDAC) activities [183]. Beyond its direct effects on cancer cells, EGCG also regulates the expression of microRNAs (miRNAs), small non-coding RNA molecules that play key roles in cellular processes such as apoptosis, growth, and metabolism, all of which are central to cancer biology. EGCG has been shown to inhibit androgen receptor activity in prostate cancer cells by modulating androgen-regulated miRNAs, such as miRNA-21 and miRNA-330, which suppress tumorigenesis [184]. Moreover, EGCG upregulates miRNAs such as miR-210, miR-29a, miR-203, and miR-125b in cervical cancer cells, further supporting its role in inhibiting cancer-cell proliferation and enhancing apoptosis [185]. EGCG also limits tumor invasion and metastasis by inhibiting MMPs such as MMP-2 and MMP-9, which are critical for prostate cancer progression [186]. Furthermore, EGCG may block urokinase-like plasminogen activator (uPA), a protease involved in cancer progression [186]. Research has also indicated that EGCG can exert antitumor effects by inhibiting glycolytic enzymes, reducing glucose metabolism, and further suppressing cancer-cell growth [187]. Additionally, EGCG’s combination with standard chemotherapy drugs may enhance their efficacy through additive or synergistic effects, while also mitigating chemotherapy-related side effects [64]. In conclusion, EGCG demonstrates significant potential not only for cancer chemoprevention but also as a therapeutic agent in cancer treatment. Its broad spectrum of biological actions, including antioxidant, antiangiogenic, and antitumor effects, underscores its therapeutic promise in cancer care, along with its broader applicability in treating various diseases [77,188].

### 3.8. EGCG in Metabolic Syndrome

Elevated blood glucose, insulin resistance, excessive body fat, especially visceral obesity, high cholesterol, hyperlipidemia, and hypertension are all risk factors that are included in the metabolic syndrome. Cardiovascular illnesses, type 2 diabetes, obesity, and renal problems are all considerably more likely to develop in people with these symptoms [189,190]. Moreover, prothrombotic states, sleep apnea, non-alcoholic fatty liver disease (NAFLD), and chronic inflammation are additional problems linked to metabolic syndrome [191]. Elevated serum uric acid (hyperuricemia) is also a known marker of metabolic syndrome and a contributor to the progression of related diseases [192,193]. With modern lifestyles characterized by diets high in processed foods, sugars, and fats, coupled with low physical activity, there has been a dramatic increase in the prevalence of metabolic disorders worldwide. This rising burden has made metabolic syndrome and its comorbidities a focal point of global research. EGCG has been studied for its potential to alleviate various aspects of metabolic syndrome. One of EGCG’s beneficial effects is its ability to lower uric acid levels in individuals with metabolic disorders by either reducing its production or enhancing its excretion. This is primarily achieved through EGCG’s inhibition of xanthine oxidase (XOD), an enzyme responsible for converting purines like hypoxanthine to xanthine, and ultimately to uric acid [194]. Additionally, EGCG may act as a preventive measure against obesity and oxidative stress by boosting energy expenditure, enhancing fat oxidation, and lowering the respiratory quotient, which in turn helps reduce body mass index (BMI) and abdominal fat [195]. These effects can be attributed to EGCG’s capacity to interact with various biological molecules, thereby influencing enzymatic activities and cellular signaling pathways that regulate metabolism. Chronic inflammation is a key feature of metabolic syndrome, driven by elevated levels of inflammatory mediators, such as cytokines and adipokines [196,197,198]. Notably, pro-inflammatory cytokines like IL-1 and IL-18 play a significant role in the formation of atherosclerotic plaques, a major complication of metabolic syndrome [199,200]. In addition, the presence of high blood glucose levels increases the amount of glucose within endothelial cells, facilitating the oxidative breakdown of glucose byproducts and triggering oxidative stress. As a result, dietary changes are considered a vital strategy in mitigating the rising prevalence of metabolic syndrome. Green tea, and especially EGCG, offers a promising approach for both preventing and managing metabolic syndrome and associated age-related diseases, either through direct dietary intake or supplementation. Clinical research has consistently shown that EGCG can help reduce body weight, BMI, and abdominal fat [201]. The antioxidant properties of EGCG also contribute to its effects on metabolic health. By modulating the expression of genes and proteins, EGCG suppresses key enzymes and transcription factors involved in fat storage and adipogenesis, such as adenosine monophosphate-activated protein kinase (AMPK) [202]. Recent studies have further revealed that EGCG’s impact extends to mitochondrial function, influencing processes like mitochondrial biogenesis, ATP production, and cell-cycle regulation, as well as promoting apoptosis in mitochondria [203,204]. Furthermore, EGCG benefits the gut microbiota, increasing the abundance of beneficial Bifidobacterium species, which improves overall energy metabolism. EGCG also stimulates enzymes involved in lipolysis and helps regulate serum urate levels, solidifying its role as a promising agent for weight management and improved lipid metabolism.

#### 3.8.1. EGCG in Insulin Resistance and Hypertension

Insulin resistance is a pathological condition characterized by the reduced responsiveness of target tissues—such as muscle, liver, and adipose tissue—to insulin. This dysfunction disrupts glucose homeostasis, impairing the body’s ability to effectively clear glucose from the bloodstream. To compensate, pancreatic β-cells increase insulin secretion, maintaining normoglycemia in the early stages, but this compensation may eventually fail, contributing to the progression of metabolic disorders. Insulin resistance is associated with a cluster of metabolic abnormalities, including hyperinsulinemia, hyperglycemia, dyslipidemia, visceral fat accumulation, obesity, hypertension, hyperuricemia, endothelial dysfunction, and heightened inflammatory responses [205,206]. One key feature of insulin resistance is the impaired ability of insulin to suppress lipolysis in adipose tissue. This leads to elevated circulating free fatty acids (FFAs), which further disrupt metabolic homeostasis by activating protein kinases in the liver, stimulating gluconeogenesis, and promoting lipid synthesis and breakdown. At the same time, these FFAs inhibit protein kinase activity in muscle tissue, reducing glucose uptake. This dysregulation exacerbates the cycle of insulin resistance by impairing lipid and glucose metabolism [207]. Recent research has identified EGCG as a promising compound for alleviating insulin resistance and improving glucose metabolism. Animal studies have demonstrated that green tea catechins can lower blood glucose levels and improve glucose tolerance, highlighting their potential in restoring metabolic balance. EGCG exerts its beneficial effects through multiple mechanisms. It reduces oxidative stress in adipocytes, enhancing insulin sensitivity and promoting glucose uptake [208]. Clinical trials have confirmed these findings; for instance, Lee et al. observed that GTE significantly reduced fasting blood glucose, insulin levels, and insulin resistance in obese patients with hypertension. Similarly, Liu et al. reported that GTE improved metabolic markers, including increased HDL cholesterol, reduced triglycerides, and elevated GLP-1 levels, which play a critical role in regulating blood glucose [209]. At a molecular level, EGCG improves insulin signaling by upregulating insulin receptor substrate-1 (IRS-1) and enhancing its phosphorylation, a critical step for efficient glucose uptake. Animal studies have also shown that EGCG improves glucose tolerance and reduces postprandial glucose spikes by delaying glucose absorption in the intestine [210,211]. A study by Lee and colleagues further demonstrated that GTE, including EGCG, exhibit hypoglycemic effects in humans, while other research confirmed reductions in plasma glucose concentrations in overweight or obese postmenopausal women when combined with regular physical exercise [211,212,213]. Collectively, these findings suggest that EGCG improves glucose homeostasis through various mechanisms, including reducing oxidative stress, enhancing insulin signaling, improving lipid profiles, and modulating glucose absorption. Given these promising results, EGCG has emerged as a potential therapeutic agent for managing insulin resistance and associated metabolic disorders. Continued research is necessary to explore its long-term efficacy and establish standardized treatment protocols.

#### 3.8.2. EGCG in Regulation of Adipose Mass and Lipid Metabolism

EGCG has attracted significant attention for its potential effects on energy regulation, appetite suppression, and lipid metabolism. Studies indicate that EGCG can reduce food intake, inhibit lipogenesis, and prevent the differentiation of preadipocytes, simultaneously promoting fat oxidation and lipolysis. These effects are largely mediated through the activation of the AMP-activated protein kinase (AMPK) pathway, a critical regulator of energy homeostasis. AMPK activation by EGCG stimulates FFA oxidation, mitigating lipid accumulation in tissues such as the liver. For example, in hepatic cells like the HepG2 cell line, EGCG has been shown to counteract lipid buildup caused by FFAs. By enhancing AMPK activity, EGCG also suppresses hepatic gluconeogenesis and promotes fat oxidation, contributing to reductions in adipose tissue mass and overall body weight [214]. Clinical evidence and systematic reviews consistently support the beneficial effects of EGCG on lipid metabolism. Asbaghi et al. reviewed studies showing that daily supplementation of GTE in doses exceeding 800 mg over at least eight weeks significantly improved lipid profiles in patients with type 2 diabetes. The review reported reductions in serum triglycerides and total cholesterol levels [215]. Similar findings have been observed in both normal-weight and obese populations, where EGCG supplementation has been linked to decreased levels of LDL cholesterol and total cholesterol [216]. A study involving healthy Japanese women further highlighted EGCG’s role in lipid regulation, demonstrating that increased plasma and urinary concentrations of green tea catechins were associated with improved plasma lipid profiles. Randomized, placebo-controlled trials in obese Taiwanese women provided additional clinical evidence, showing that 12 weeks of GTE supplementation led to significant decreases in total cholesterol, LDL cholesterol, and triglyceride levels, alongside increases in HDL and adiponectin levels [217,218]. Moreover, EGCG’s combination with caffeine has shown synergistic benefits on metabolic health. This combination influenced gut microbiota composition, increasing populations of beneficial bacteria such as Bifidobacterium and enhancing the production of short-chain fatty acids (SCFAs) in feces, as observed in animal models. These changes further underscore the role of EGCG in promoting metabolic balance [219]. Choi et al. provided additional insights into EGCG’s mechanisms, showing that it stimulates lipolysis and suppresses lipogenesis in adipocytes through AMPK activation. EGCG also facilitates visceral fat reduction by inducing autophagy and promoting lipolysis in white adipose tissue, again mediated by the AMPK pathway. These findings collectively highlight EGCG’s potential as a therapeutic agent for managing obesity and dyslipidemia. Its ability to modulate lipid metabolism, reduce adipose tissue mass, and improve cholesterol and triglyceride levels underscores its promise in addressing metabolic disorders. EGCG emerges as a potent candidate for combating obesity and improving lipid profiles, offering a multifaceted approach to metabolic disease management.

#### 3.8.3. EGCG in Hyperuricemia and Uric Acid Metabolism

Uric acid, the end product of purine metabolism, is generated through the breakdown of both endogenous and dietary purines [220,221]. Elevated levels of uric acid in the blood, known as hyperuricemia, are linked to various metabolic disorders, including insulin resistance, type 2 diabetes, coronary heart disease, chronic kidney disease, gout, and NAFLD [222]. Maintaining serum urate levels below 7 mg/dL in men and 6 mg/dL in women is critical for preventing the development of hyperuricemia and associated conditions, such as cardiovascular diseases and gout [223,224]. Current treatments for hyperuricemia, including xanthine oxidase inhibitors (such as allopurinol, febuxostat) and uricosuric agents, have limitations, including side effects like kidney stones, liver damage, and hypersensitivity reactions, which make them unsuitable for all patients [224,225]. As a result, there is growing interest in alternative or complementary therapies, such as EGCG, which may help manage hyperuricemia and related metabolic conditions. Several studies have demonstrated that EGCG plays a significant role in promoting the excretion of uric acid. This effect is achieved by regulating uric acid transporters in the kidneys, including those involved in uric acid secretion and reabsorption. Specifically, EGCG has been shown to enhance the expression of organic anion transporters (OAT1 and OAT3) and organic cation transporter 1 (OCT1), which are involved in uric acid secretion, while downregulating the expression of reabsorption transporters such as urate transporter 1 (URAT1) and glucose transporter 9 (GLUT9) [226,227]. In animal models of hyperuricemia, EGCG promoted the activity of OAT1 and reduced the expression of GLUT9 in kidney tissues, leading to enhanced uric acid excretion [194]. Furthermore, EGCG has been reported to reduce serum uric acid levels by inhibiting enzymes involved in purine metabolism, particularly xanthine oxidase (XOD) and adenosine deaminase (ADA) [228]. EGCG inhibits up to 80% of XOD activity by binding to the flavin adenine dinucleotide (FAD) site of the enzyme, thereby preventing it from catalyzing the conversion of hypoxanthine to xanthine and ultimately to uric acid [229]. Both in vitro and in vivo studies have confirmed that EGCG significantly reduces serum uric acid levels, decreases XOD activity, and promotes the excretion of purine metabolites such as xanthine and hypoxanthine via urine. This evidence highlights EGCG as a potential natural agent for managing hyperuricemia and its related diseases. The ability of EGCG to modulate uric acid metabolism through multiple mechanisms, including the inhibition of XOD activity and the regulation of uric acid transporters, positions it as a promising therapeutic option for the prevention and management of hyperuricemia and its associated conditions. These findings suggest that EGCG may offer a safe and effective alternative or adjunct to conventional pharmacological treatments for hyperuricemia.

## 4. EGCG in Human Diseases

### 4.1. Effect of EGCG on Type 2 Diabetes Mellitus

Diabetes mellitus is a group of metabolic disorders marked by chronic hyperglycemia, which results from abnormalities in insulin secretion, insulin action, or both [230]. In type 2 diabetes, insulin resistance—where target tissues, including skeletal muscle, adipose tissue, and liver, become less responsive to insulin—plays a central role in disrupting glucose homeostasis [231]. This form of diabetes is often associated with complications such as cardiovascular disease, chronic kidney disease, and diabetic retinopathy [205,231]. While several medications, including insulin and non-insulin treatments (e.g., β-glucosidase inhibitors, GLP-1 receptor agonists, and insulin-sensitizing agents), are commonly used to manage type 2 diabetes, they can lead to adverse effects, such as hypoglycemia, weight gain, gastrointestinal issues, and cardiovascular risks [232,233]. Additionally, the management of diabetes requires personalized treatment plans to address comorbidities and individual responses, adding complexity to care. As a result, there is growing interest in exploring natural compounds like EGCG as potential alternatives or adjuncts to conventional therapies for diabetes prevention and management. EGCG has gained attention for its potential to improve metabolic control in individuals with diabetes. A randomized, double-blind, placebo-controlled clinical trial revealed that EGCG supplementation improved insulin sensitivity, glycemic control, and lipid profiles in individuals with type 2 diabetes. Notably, it reduced serum triglycerides and total cholesterol while increasing HDL cholesterol and glucagon-like peptide-1 (GLP-1) levels, suggesting a favorable effect on both glucose and lipid metabolism [209]. EGCG has also been shown to protect pancreatic β-cells from oxidative damage and apoptosis, thereby enhancing insulin secretion and sensitivity. This effect is believed to be partly due to the suppression of microRNA (miR-16-5p), which regulates pancreatic β-cell function [234]. Additionally, EGCG-derived autoxidation products help improve insulin sensitivity by reducing liver-derived secretory selenoprotein P (SELENOP), a protein involved in insulin resistance [235]. The antioxidant and anti-inflammatory properties of EGCG further support its role in managing type 2 diabetes. EGCG decreases oxidative stress by lowering the production of ROS and reducing the expression of iNOS, which contributes to vascular dysfunction in diabetic individuals. In a clinical study involving 50 diabetic patients, daily EGCG supplementation (300 mg for eight weeks) resulted in significant improvements in fasting blood glucose, body weight, and high-sensitivity C-reactive protein (hs-CRP) levels, suggesting reductions in both hyperglycemia and inflammation. Additionally, EGCG has been found to target NLRP3 inflammasomes, which are activated in response to high-fat diets and contribute to chronic inflammation and insulin resistance. By suppressing NLRP3 activation, EGCG may improve insulin signaling and glucose tolerance, providing further evidence of its potential to alleviate diabetes symptoms [236]. EGCG’s multifaceted effects on type 2 diabetes can be summarized in several key actions. It enhances insulin synthesis, with supplementation shown to significantly increase insulin production compared to control groups [70]. EGCG also acts on various steps of the insulin signaling pathway, improving β-cell function and protecting these cells from apoptosis induced by inflammatory cytokines, thus supporting sustained insulin production [237]. Another important action is EGCG’s ability to reduce insulin resistance, particularly in skeletal muscle, where excess fatty acids impair glucose uptake. By alleviating this resistance, EGCG facilitates better glucose metabolism and utilization [152]. Its potent antioxidant activity further enhances insulin sensitivity by reducing oxidative stress, a key contributor to insulin resistance [238]. In addition, EGCG suppresses lipid accumulation in tissues, primarily through the activation of the AMPK/ACC pathway, which helps prevent the storage of excess fat and the resulting metabolic dysfunction [238,239]. EGCG also promotes glucose uptake in response to insulin, which enhances glucose homeostasis and supports better blood sugar control [155,156]. Finally, EGCG helps reduce markers of stress and inflammation, which are involved in the development of insulin resistance. This reduction in metabolic and inflammatory stress supports better insulin function and improves the body’s ability to manage glucose effectively [240]. These comprehensive effects position EGCG as a promising natural agent for managing type 2 diabetes and enhancing metabolic health, offering a potential alternative or adjunct to conventional pharmaceutical treatments.

### 4.2. Effect of EGCG on Obesity

Obesity is a complex global health issue marked by metabolic imbalances, chronic inflammation, and oxidative stress, all of which contribute to severe health complications. It arises primarily from an energy imbalance between caloric intake and expenditure, resulting in excessive fat accumulation, adipocyte hypertrophy, and dysfunction in adipose tissue [241]. Obesity is closely linked to a wide range of conditions, including type 2 diabetes, cardiovascular diseases, hypertension, insulin resistance, dyslipidemia, and cognitive decline. Furthermore, it exacerbates systemic inflammation and impairs immune function, increasing susceptibility to viral infections [219,241]. Emerging research in human and animal models highlights the potential role of compounds found in green tea, particularly EGCG, in mitigating obesity and its associated metabolic disturbances. Studies suggest that EGCG enhances thermogenesis and fat oxidation, contributing to reductions in body fat and improvements in metabolic health. These findings position EGCG as a promising intervention for preventing and managing obesity. EGCG supplementation has demonstrated several benefits in addressing obesity-related biomarkers. In one clinical trial, eight weeks of EGCG supplementation led to significant reductions in fasting plasma triglyceride levels, blood pressure, and serum kisspeptin concentrations [242]. A meta-analysis of randomized controlled trials further revealed that daily consumption of green tea, in doses of less than 500 mg, significantly reduced body weight, BMI, and waist circumference over 12 weeks [243]. Additional studies have reported decreases in triglyceride levels, visceral fat percentages, and the LDL/HDL cholesterol ratio following EGCG intake, reinforcing its role in improving metabolic health and reducing body fat [244]. The anti-obesity effects of EGCG are mediated through several mechanisms. For instance, EGCG enhances insulin sensitivity by modulating key pathways, such as the IRS-1 and ERK/cAMP response element-binding protein (CREB)/brain-derived neurotrophic factor (BDNF) signaling cascades, which are essential for maintaining glucose homeostasis and supporting cognitive function [210]. Moreover, EGCG has been shown to suppress the JAK2/STAT3 pathway, thereby reducing the release of pro-inflammatory cytokines such as TNF-α, IL-1, and IL-6 in response to inflammatory stimuli like palmitic acid [245]. In addition to its anti-inflammatory properties, EGCG also inhibits pancreatic lipase, a critical enzyme involved in dietary fat digestion, thereby reducing fat absorption and promoting fat breakdown [246]. This combination of enhanced fat oxidation, decreased fat absorption, and improved insulin sensitivity underscores EGCG’s potential as a natural and effective agent in combating obesity. In summary, EGCG and GTE represent promising additions to weight management strategies. Their ability to regulate lipid metabolism, suppress inflammation, and improve metabolic markers supports their use in promoting long-term metabolic health and preventing obesity-related disorders. Incorporating EGCG into dietary or therapeutic approaches may provide significant benefits in the global effort to manage and prevent obesity.

### 4.3. Cardioprotective Effects of EGCG

Cardiovascular diseases (CVDs) encompass a wide range of conditions affecting the heart and blood vessels, including coronary artery disease, peripheral arterial disease, heart failure, cerebrovascular disease, and congenital heart defects. A key factor in the progression of CVDs is endothelial dysfunction, which impairs the endothelium’s ability to regulate vascular tone, coagulation, blood flow, and permeability. This dysfunction significantly contributes to the development of atherosclerosis, a condition characterized by lipid accumulation and inflammation within arterial walls, leading to arterial narrowing and stiffness. Atherosclerosis is a major cause of cardiovascular events such as heart attacks and strokes and is often associated with metabolic disorders like type 2 diabetes [247]. CVDs remain a leading cause of global mortality, accounting for nearly one-third of all deaths worldwide [137,138]. Emerging evidence indicates that the regular consumption of tea, particularly green tea, is associated with a reduced risk of CVDs. Numerous studies have reported an inverse relationship between tea intake and cardiovascular events, with green tea polyphenols such as EGCG playing a central role in these protective effects [248]. EGCG exerts multiple cardioprotective effects (Table 1). It reduces LDL cholesterol, decreases oxidative stress by neutralizing ROS, and lowers inflammatory markers such as TNF-α [249]. EGCG also improves endothelial function, promotes arterial dilation, and inhibits myeloperoxidase, an enzyme linked to oxidative damage and inflammation. In clinical studies, EGCG has demonstrated its ability to lower both systolic and diastolic blood pressure in individuals with prehypertension or type 2 diabetes, further supporting its cardiovascular benefits. Additionally, regular green tea consumption has been shown to enhance serum antioxidant capacity, reduce oxidative stress, and improve blood pressure regulation, all of which contribute to cardiovascular health [250]. Animal studies have found that EGCG mitigates salt-induced hypertension and prevents renal damage, highlighting its potential for managing hypertension and related complications. In obese hypertensive patients, three months of GTE supplementation resulted in significant reductions in insulin resistance, inflammation, and oxidative stress—key contributors to CVD progression. The cardiovascular benefits of EGCG are attributed to its ability to improve insulin sensitivity, reduce leukocyte adhesion to the endothelium, and suppress inflammatory signaling pathways such as NF-κB, which regulates the production of inflammatory cytokines and adhesion molecules [250,251]. Furthermore, EGCG prevents LDL cholesterol oxidation, inhibits cholesterol absorption in the intestine, suppresses cholesterol synthesis, and reduces vascular smooth-muscle-cell proliferation. These effects collectively decrease the risk of atherosclerosis and plaque formation [252]. Emerging research in the field of epigenetics has unveiled additional mechanisms by which EGCG may influence cardiovascular health. EGCG acts as an epigenetic modulator, impacting the activity of enzymes involved in gene expression regulation, such as DNMT and histone acetyltransferase (HAT). These enzymes play key roles in DNA methylation and histone acetylation, which influence inflammatory and metabolic gene expression. EGCG has been shown to enhance autophagy, improve endothelial function, and regulate inflammatory responses through the activation of AMPK and mTOR signaling pathways. Furthermore, it induces histone H3 hypoacetylation, reduces the expression of HDAC1 in endothelial cells, and downregulates pro-inflammatory mediators [253,254]. In summary, EGCG offers a multifaceted approach to cardiovascular protection, targeting traditional cardiovascular risk factors like LDL cholesterol, oxidative stress, and blood pressure, while also modulating epigenetic processes that influence endothelial function and inflammation. These findings highlight the potential of EGCG as a promising therapeutic agent for preventing and managing CVDs through its broad-spectrum effects on vascular health, lipid metabolism, and gene regulation. Table 1 reports on the main effects of EGCG in CVDs.

### 4.4. Neuroprotective Effects of EGCG

Neural tissue is particularly vulnerable to oxidative damage due to its high metabolic activity, substantial lipid content, and relatively low antioxidant defenses. The brain’s structure, coupled with its significant oxygen consumption, predisposes it to damage caused by ROS and RNS. These reactive molecules contribute to cellular dysfunction and neurodegeneration. In this context, catechins, especially EGCG from green tea, have gained attention for their neuroprotective properties. EGCG can cross the blood–brain barrier and exert strong antioxidant effects within the central nervous system (CNS). By scavenging ROS and RNS, EGCG neutralizes these harmful molecules and prevents oxidative injury. In addition to its antioxidant capabilities, EGCG chelates transition metal ions such as Cu^2+^, Zn^2+^, and Fe^2+^, which are involved in generating free radicals through Fenton chemistry. By reducing the availability of these metal ions, EGCG minimizes oxidative damage and protects neural tissues. For instance, studies have shown that EGCG effectively prevents iron-induced DNA damage and lipid peroxidation in brain mitochondria, which are highly susceptible to oxidative stress [30,255]. Furthermore, EGCG enhances the activity and expression of crucial antioxidant enzymes, including superoxide dismutase (SOD), catalase, glutathione peroxidase, and glutathione reductase. These enzymes maintain cellular redox balance and safeguard neurons against oxidative damage. EGCG also inhibits pro-oxidative enzymes such as monoamine oxidase (MAO)-B and nitric oxide synthase (NOS), reducing ROS generation and curbing processes that drive neurodegeneration [256]. Neurodegenerative diseases like Alzheimer’s, Parkinson’s, and Huntington’s disease are characterized by the accumulation of misfolded proteins, including amyloid beta (Aβ) and α-synuclein, which aggregate into toxic forms and disrupt cellular function. EGCG has demonstrated the ability to interact with these misfolded proteins, preventing the formation of toxic aggregates and promoting the development of non-toxic oligomers. This mechanism is particularly significant in mitigating the accumulation of amyloid plaques in Alzheimer’s disease and Lewy bodies in Parkinson’s disease, hallmark features of these conditions [257,258,259,260]. Oxidative stress further exacerbates protein misfolding and aggregation by destabilizing proteins and impairing cellular antioxidant defenses, thereby accelerating neurodegenerative processes. EGCG counters these effects through its potent antioxidant activity, reducing the probability of protein misfolding and limiting the damaging impact of protein aggregates [261]. Additionally, EGCG inhibits the conversion of nitrates and peroxynitrite to NO, thereby protecting neurons from ischemic damage and neurodegenerative changes [262]. EGCG also exhibits strong anti-inflammatory properties, further enhancing its neuroprotective role. Microglia, the CNS’s resident immune cells, play a central role in neuroinflammation, which is implicated in the progression of neurodegenerative diseases. EGCG suppresses microglial activation and decreases the production of pro-inflammatory cytokines, reducing neuroinflammation and shielding neurons from inflammatory damage. Moreover, EGCG protects against CNS injury caused by environmental factors like infrasound, which can aggravate neuroinflammation and lead to neurological dysfunction [256]. In summary, EGCG provides robust neuroprotection through its diverse mechanisms: neutralizing oxidative stress, chelating metal ions, inhibiting protein aggregation, and modulating neuroinflammation (Table 2). These comprehensive effects underscore EGCG’s potential as a therapeutic agent for neurodegenerative diseases where oxidative stress, protein misfolding, and inflammation are key drivers of disease progression. Further research into the molecular pathways influenced by EGCG may pave the way for novel interventions targeting these debilitating conditions.

## 5. Conclusions

Green tea has long been associated with a range of health benefits, largely due to its richness in polyphenols. Of these, EGCG stands out as the most abundant and bioactive, accounting for roughly 80% of the catechins. EGCG is of particular interest in medicinal chemistry due to its powerful biological activities and therapeutic potential.

Extensive research over the past few decades has highlighted the numerous health-promoting properties of green tea and its polyphenolic compounds. EGCG, in particular, has been the focus of a wide array of basic and clinical studies, demonstrating its potential as a therapeutic agent for various conditions. However, challenges such as its poor intestinal absorption and chemical instability continue to hinder its broader application for therapeutic or preventive purposes. Advances in nanotechnology, particularly the encapsulation of EGCG in nanocarriers, have significantly improved its stability and enhanced its bioactivity, making it a more viable option for medical use.

EGCG is non-toxic and has no reported side effects, which further enhances its appeal as a natural compound for research in disease prevention and treatment. The findings from this review suggest that EGCG regulates a wide array of cellular pathways, which could be beneficial for preventing, managing, or even reversing the progression of chronic diseases such as cardiovascular diseases, neurodegenerative disorders, diabetes, and cancer. Additionally, there is promising evidence that combining EGCG with other antioxidants may further amplify its therapeutic effects, as these compounds work synergistically to enhance their bioactivity.

Natural compounds like EGCG represent a vast, largely untapped reservoir of biologically active molecules with huge potential for medical applications. As research continues to unravel the molecular mechanisms behind EGCG’s diverse beneficial effects, it will become increasingly important to explore and understand these pathways. This knowledge will facilitate the development of novel nutraceuticals and therapeutic strategies, positioning EGCG as a promising candidate in the prevention and treatment of different human diseases.

## Figures and Tables

**Table 1:** Cardioprotective effects of EGCG.

Effect of EGCG	Description	Mechanism	References
Reduction in LDL cholesterol	Lowers LDL levels, preventing the formation of atherosclerotic plaques	Inhibits LDL oxidation, reduces intestinal cholesterol absorption, and suppresses cholesterol synthesis	[248]
Improvement of endothelial function	Promotes arterial dilation and improves blood flow regulation	Neutralizes ROS, reduces oxidative stress, lowers inflammatory markers like TNF-α, and inhibits myeloperoxidase	[251]
Reduction in oxidative stress	Increases serum antioxidant capacity and reduces oxidative damage	Neutralizes reactive oxygen species (ROS) and enhances antioxidant defense	[250]
Blood pressure reduction	Lowers systolic and diastolic blood pressure, improving cardiovascular control	Mitigates hypertension through antioxidant and anti-inflammatory effects	[250,251]
Reduction in inflammation	Decreases inflammatory markers (e.g., TNF-α) and suppresses inflammatory pathways	Regulates NF-κB pathways, reducing cytokine and adhesion molecule production	[249,251]
Improvement of insulin sensitivity	Enhances glucose management and reduces insulin resistance in obese or diabetic patients	Modulates endothelial function, improves lipid metabolism, and reduces inflammation	[250,251]
Inhibition of cellular proliferation	Reduces vascular smooth muscle cell proliferation, preventing arterial narrowing	Suppresses processes related to pathological cell growth	[252]
Epigenetic effects	Modulates gene expression and inflammatory responses through epigenetic modifications	Influences DNMT, HAT, activates AMPK and mTOR pathways, regulates DNA methylation and histone acetylation	[253,254]
Promotion of autophagy	Enhances cellular turnover and protects endothelial cells from chronic stress	Activates signaling pathways such as AMPK and mTOR	[253,254]

**Table 2:** Neuroprotective effects of EGCG.

Effect of EGCG	Description	Mechanism	References
Antioxidant activity	Neutralization of ROS/RNS	-Scavenging of reactive oxygen and nitrogen species. -Chelation of metal ions (Cu^2+^, Zn^2+^, Fe^2+^) to reduce free radical formation. -Protection against iron-induced oxidative damage. -Enhancement of antioxidant enzyme activity (SOD, catalase, glutathione peroxidase, glutathione reductase). -Inhibition of pro-oxidative enzymes (MAO-B, NOS).	[255,256]
Inhibition of protein aggregation	Prevention of toxic aggregate formation	-Interaction with misfolded proteins (amyloid beta, α-synuclein). -Inhibition of toxic aggregation and facilitation of non-toxic oligomer formation. -Prevention of amyloid plaque and Lewy body accumulation.	[257,258]
Reduction in oxidative stress related to protein misfolding	Mitigation of damage associated with ROS/RNS	-Reduction in the oxidative stress promoting protein misfolding. -Inhibition of the conversion of nitrates and peroxynitrite into nitric oxide, preventing ischemic neuronal damage.	[259,262]
Modulation of neuroinflammation	Reduction in microglial activation and inflammation	-Inhibition of microglial activation and production of pro-inflammatory cytokines. -Protection against neuroinflammatory damage induced by environmental factors (e.g., infrasound).	[256]

## Abbreviations

The following abbreviation are used in this manuscript:

ADA	Adenosine deaminase
AMPK	AMP-activated protein kinase
BMI	Body mass index
CNS	Central nervous system
COMT	Catechol-*O*-methyltransferase
EC	(–)-Epicatechin
ECG	(–)-Epicatechin gallate
EGC	(–)-Epigallocatechin
EGCG	(–)-Epigallocatechin gallate
FAD	Flavin adenine dinucleotide
FFAs	Free fatty acids
GC	(+)-Gallocatechin
GLUT9	Glucose transporter 9
HDAC	Histone deacetylase
HGF	Hepatocyte growth factor
HNF4α	Hepatocyte nuclear factor 4α
HSV	Herpes simplex virus
IAV	Influenza A
IFN-λ1	Interferon lambda 1
IRS-1	Insulin receptor substrate-1
LDL	Low-density lipoprotein
MAO-B	Monoamine oxidase-B
MRSA	Methicillin-resistant Staphylococcus aureus
NAFLD	Non-alcoholic fatty liver disease
NO	Nitric oxide
NOAEL	No-observed-adverse-effect level
NOS	Nitric oxide synthase
OAT1	Organic anion transporter 1
OAT3	Organic anion transporter 3
OCT1	Organic cation transporter 1
RIG-I	Retinoic acid-inducible gene I
ROS	Reactive oxygen species
RNS	Reactive nitrogen species
SULT	Sulfotransferase
TLA3	Toll-like receptor 3
uPA	Urokinase plasminogen activator
VSMCs	Vascular smooth muscle cells
XOD	Xanthine oxidase
